# ﻿Species diversity, molecular phylogeny and ecological habits of *Cyanosporus* (Polyporales, Basidiomycota) with an emphasis on Chinese collections

**DOI:** 10.3897/mycokeys.86.78305

**Published:** 2022-01-11

**Authors:** Shun Liu, Tai-Min Xu, Chang-Ge Song, Chang-Lin Zhao, Dong-Mei Wu, Bao-Kai Cui

**Affiliations:** 1 Institute of Microbiology, School of Ecology and Nature Conservation, Beijing Forestry University, Beijing 100083, China Beijing Forestry University Beijing China; 2 College of Biodiversity Conservation, Southwest Forestry University, Kunming 650224, China Southwest Forestry University Kunming China; 3 Biotechnology Research Institute, Xinjiang Academy of Agricultural and Reclamation Sciences / Xinjiang Production and Construction Group Key Laboratory of Crop Germplasm Enhancement and Gene Resources Utilization, Shihezi, Xinjiang 832000, China Biotechnology Research Institute, Xinjiang Academy of Agricultural and Reclamation Sciences Shihezi China

**Keywords:** brown-rot fungi, distribution areas, host trees, multi-gene phylogeny, new species

## Abstract

*Cyanosporus* is a genus widely distributed in Asia, Europe, North America, South America and Oceania. It grows on different angiosperm and gymnosperm trees and can cause brown rot of wood. Blue-tinted basidiomata of *Cyanosporus* makes it easy to distinguish from other genera, but the similar morphological characters make it difficult to identify species within the genus. Phylogeny and taxonomy of *Cyanosporus* were carried out based on worldwide samples with an emphasis on Chinese collections, and the species diversity of the genus is updated. Four new species, *C.flavus*, *C.rigidus*, *C.subungulatus* and *C.tenuicontextus*, are described based on the evidence of morphological characters, distribution areas, host trees and molecular phylogenetic analyses inferred from the internal transcribed spacer (ITS) regions, the large subunit of nuclear ribosomal RNA gene (nLSU), the small subunit of nuclear ribosomal RNA gene (nSSU), the small subunit of mitochondrial rRNA gene (mtSSU), the largest subunit of RNA polymerase II (RPB1), the second largest subunit of RNA polymerase II (RPB2), and the translation elongation factor 1-α gene (TEF). Our study expanded the number of *Cyanosporus* species to 35 around the world including 23 species from China. Detailed descriptions of the four new species and the geographical locations of the *Cyanosporus* species in China are provided.

## ﻿Introduction

*Cyanosporus* was proposed as a monotypic genus for *Polyporuscaesius* (Schrad.) Fr. based on its cyanophilous basidiospores (McGinty 1909). However, *Tyromycescaesius* (Schrad.) Murrill and *Postiacaesia* (Schrad.) P. Karst. were frequently used instead of *Cyanosporuscaesius* (Schrad.) McGinty in subsequent studies (Donk 1960; Jahn 1963; Lowe 1975). Later, four species in the *Postiacaesia* complex were described from Europe, viz., *P.luteocaesia* (A. David) Jülich, *P.subcaesia* (A. David) Jülich, *P.alni* Niemelä & Vampola and *P.mediterraneocaesia* M. Pieri & B. Rivoire (David 1974, 1980; Jahn 1979; Pieri and Rivoire 2005). Then, the subgenus Cyanosporus (McGinty) V. Papp was proposed for the species of *P.caesia* complex (Papp 2014). Miettinen et al. (2018) revised the species concept of the *P.caesia* complex based on morphology and two gene markers (ITS and TEF) and raised the species number of the complex to 24, including six species from China.

Previously, species identification of the *P.caesia* complex was only based on morphological characters and host trees in China, and only two species were recorded from China before Dai (2012), viz., *P.alni* and *P.caesia*. Recently, taxonomic studies of *P.caesia* complex in China have been carried out, and some new species have been described based on both morphological characteristics and molecular data. Shen et al. (2019) carried out a comprehensive study on *Postia* and related genera, in which *Cyanosporus* was supported as an independent genus with 12 species were accepted in this genus. Liu et al. (2021a) studied the species diversity and molecular phylogeny of *Cyanosporus* and the number of *Cyanosporus* species was expanded to 31 around the world, including 19 species from China. These studies have greatly enriched the species of *Cyanosporus* in China. Currently, the morphological characteristics of the genus are as follows: basidiomata annual, pileate or resupinate to effused-reflexed, soft corky, corky to fragile. Pileal surface white to cream to greyish brown, usually with blue tint. Pore surface white to cream, frequently bluish; pores round to angular. Context white to cream, corky. Tubes cream, fragile. Hyphal system monomitic; generative hyphae clamped, IKI–, CB–. Cystidia usually absent, cystidioles occasionally present. Basidiospores narrow, allantoid to cylindrical, hyaline, usually slightly thick-walled, smooth, IKI–, weakly CB+.

**Figure 1.**
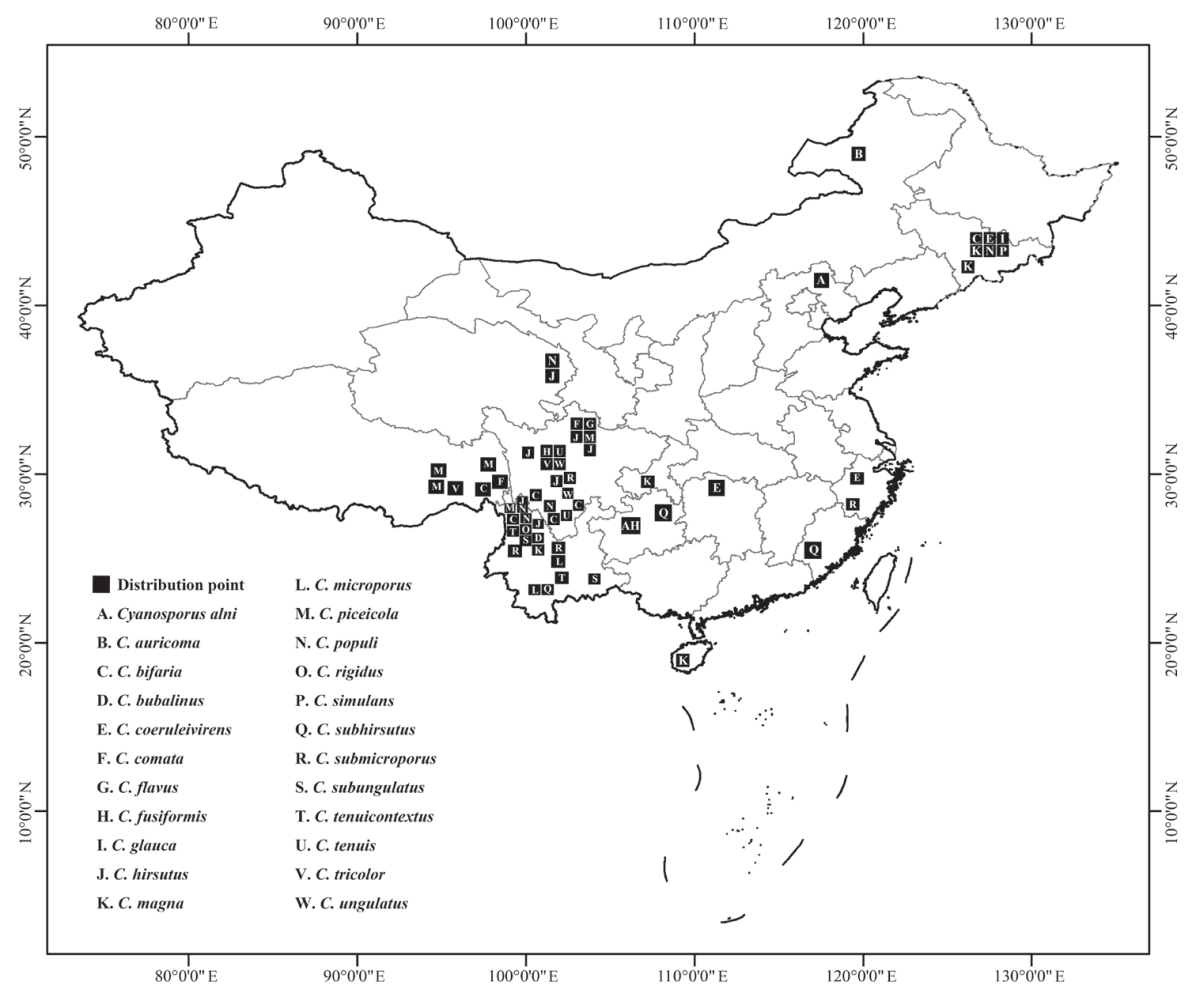
The geographical locations of the *Cyanosporus* species distributed in China.

*Cyanosporus* species usually have blue-tinted basidiomata, which makes it easy to recognize. Some specimens with blue-tinted basidiomata were collected during investigations into the diversity of polypores in China, and four undescribed species of *Cyanosporus* were discovered. To confirm the affinity of the undescribed species to *Cyanosporus*, phylogenetic analyses were carried out based on the combined datasets of ITS+TEF and ITS+nLSU+nSSU+mtSSU+RPB1+RPB2+TEF sequences. During the investigation and study of *Cyanosporus*, the information of host trees and distribution areas of species in the genus from China were also obtained (Table 1). Four new species are described and illustrated in the current study, and the geographical locations of the *Cyanosporus* species distributed in China are indicated on the map (Fig. 1).

**Table 1.** The main ecological habits of *Cyanosporus* with an emphasis on distribution areas and host trees. New species are shown in bold.

Species	Distribution in the world	Distribution in China	Climate zone	Host	Reference
*C.alni* (Niemelä & Vampola) B.K. Cui, L.L. Shen & Y.C. Dai	Europe (Czech Republic, Denmark, Finland, Germany, Norway, Poland, Russia, Slovakia), East Asia (China)	Guizhou, Hebei	Temperate	Angiosperm (*Alnus*, *Betula*, *Corylus*, *Fagus*, *Populus*, *Quercus*)	Miettinen et al. 2018; present study
*C.arbuti* (Spirin) B.K. Cui & Shun Liu	North America (USA)		Temperate	Angiosperm (*Arbutus*)	Miettinen et al. 2018
*C.auricomus* (Spirin & Niemelä) B.K. Cui & Shun Liu	Europe (Finland, Poland, Russia), East Asia (China)	Inner Mongolia	Temperate to boreal	Gymnosperm (*Pinus*, *Picea*)	Miettinen et al. 2018; Liu et al. 2021a
*C.bifarius* (Spirin) B.K. Cui & Shun Liu	Europe (Russia), East Asia (China, Japan)	Jilin, Sichuan, Yunnan	Cold temperate	Gymnosperm (*Picea*, *Pinus*, *Larix*)	Miettinen et al. 2018; present study
*C.bubalinus* B.K. Cui & Shun Liu	East Asia (China)	Yunnan	Temperate	Gymnosperm (*Pinus*)	Liu et al. 2021a
*C.caesiosimulans* (G.F. Atk.) B.K. Cui & Shun Liu	Europe (Finland, Russia), North America (USA)		Temperate	Angiosperm (*Corylus*, *Fagus*, *Populus*) and gymnosperm (*Abies*, *Picea*)	Miettinen et al. 2018
*C.caesius* (Schrad.) McGinty	Europe (Czech Republic, Denmark, Finland, France, Germany, Russia, Slovakia, Spain, UK)		Common in temperate, rare in south boreal zone	Angiosperm (*Betula*, *Fagus*, *Salix*) and gymnosperm (*Abies*, *Picea*)	Miettinen et al. 2018
*C.coeruleivirens* (Corner) B.K. Cui, Shun Liu & Y.C. Dai	Asia (China, Indonesia), Europe (Russia)	Hunan, Jilin, Zhejiang	Warm temperate	Angiosperm (*Tilia*, *Ulmus*)	Miettinen et al. 2018; present study
*C.comatus* (Miettinen) B.K. Cui & Shun Liu	North America (USA), East Asia (China)	Sichuan, Xizang	Temperate	Angiosperm (*Acer*) and gymnosperm (*Abies*, *Picea*, *Tsuga*)	Miettinen et al. 2018; present study
*C.cyanescens* (Miettinen) B.K. Cui & Shun Liu	Europe (Estonia, Finland, France, Poland, Russia, Spain, Sweden)		Temperate to Mediterranean mountains	Gymnosperm (*Abies*, *Picea*, *Pinus*)	Miettinen et al. 2018
***C.flavus* B.K. Cui & Shun Liu**	**East Asia (China)**	**Sichuan**	**Plateau humid climate**	**Gymnosperm (*Abies*, *Picea*)**	**Present study**
*C.fusiformis* B.K. Cui, L.L. Shen & Y.C. Dai	East Asia (China)	Guizhou, Sichuan	North temperate to subtropical	Angiosperm (*Rhododendron*)	Shen et al. 2019
*C.glauca* (Spirin & Miettinen) B.K. Cui & Shun Liu	East Asia (China), Europe (Russia)	Jilin	Cold temperate mountains	Gymnosperm (*Abies*, *Picea*)	Miettinen et al. 2018
*C.gossypinus* (Moug. & Lév.) B.K. Cui & Shun Liu	Europe (France)		Temperate	Gymnosperm (*Cedrus*)	Miettinen et al. 2018
*C.hirsutus* B.K. Cui & Shun Liu	East Asia (China)	Qinghai, Sichuan, Yunnan	Temperate to plateau continental climate	Gymnosperm (*Abies*, *Picea*)	Liu et al. 2021a; present study
*C.livens* (Miettinen & Vlasák) B.K. Cui & Shun Liu	North America (Canada, USA)		Temperate	Angiosperm (*Acer*, *Betula*, *Fagus*) and gymnosperm (*Abies*, *Larix*, *Picea*, *Tsuga*)	Miettinen et al. 2018
*C.luteocaesius* (A. David) B.K. Cui, L.L. Shen & Y.C. Dai	Europe (France)		Mediterranean	Gymnosperm (*Pinus*)	Miettinen et al. 2018
*C.magnus* (Miettinen) B.K. Cui & Shun Liu	East Asia (China)	Chongqin, Jilin, Hainan, Yunnan	Temperate	Angiosperm (*Populus*) and gymnosperm (*Cunninghamia*)	Miettinen et al. 2018; present study
*C.mediterraneocaesius* (M. Pieri & B. Rivoire) B.K. Cui, L.L. Shen & Y.C. Dai	Europe (France, Spain)		Warm temperate to Mediterranean	Angiosperm (*Buxus*, *Erica*, *Populus*, *Quercus*) and gymnosperm (*Cedrus*, *Juniperus*, *Pinus*)	Miettinen et al. 2018
*C.microporus* B.K. Cui, L.L. Shen & Y.C. Dai	East Asia (China)	Yunnan	subtropical	Angiosperm (undetermined)	Shen et al. 2019
*C.nothofagicola* B.K. Cui, Shun Liu & Y.C. Dai	Oceania (Australia), South America (Argentina)		Temperate marine climate	Angiosperm (*Nothofagus*)	Liu et al. 2021a
*C.piceicola* B.K. Cui, L.L. Shen & Y.C. Dai	East Asia (China)	Sichuan, Xizang, Yunnan	Warm temperate to subtropical	Gymnosperm (*Picea*)	Shen et al. 2019
*C.populi* (Miettinen) B.K. Cui & Shun Liu	East Asia (China), Europe (Finland, Norway, Poland, Russia), North America (USA)	Qinghai, Jilin, Sichuan, Yunnan	Boreal to temperate	Angiosperm (*Acer*, *Alnus*, *Betula*, *Populus*, *Salix*) and gymnosperm (*Picea*)	Miettinen et al. 2018; Liu et al. 2021a; present study
***C.rigidus* B.K. Cui & Shun**	**East Asia (China)**	**Yunnan**	**Warm temperate**	**Gymnosperm (*Picea*)**	**Present study**
*C.simulans* (P. Karst.) B.K. Cui & Shun Liu	East Asia (China), Europe (Estonia, Finland, France, Germany, Norway, Russia), North America (Canada, USA)	Jilin	Warm temperate to boreal	Angiosperm (*Corylus*, *Fagus*, *Populus*, *Sorbus*, *Ulmus*) and gymnosperm (*Abies*, *Cedrus*, *Juniperus*, *Picea*, *Pinus*, *Thuja*, *Tsuga*)	Miettinen et al. 2018
*C.subcaesius* (A. David) B.K. Cui, L.L. Shen & Y.C. Dai	Europe (Czech Republic, Finland, France, Russia, UK)		Temperate	Angiosperm (*Alnus*, *Carpinus*, *Crataegus*, *Corylus*, *Fagus*, *Fraxinus*, *Malus*, *Populus*, *Prunus*, *Quercus*, *Salix*, *Ulmus*)	Miettinen et al. 2018
*C.subhirsutus* B.K. Cui, L.L. Shen & Y.C. Dai	East Asia (China)	Guizhou, Fujian, Yunnan	Warm temperate to subtropical	Angiosperm (*Pterocarya*)	Shen et al. 2019
*C.submicroporus* B.K. Cui & Shun Liu	East Asia (China)	Sichuan, Yunnan, Zhejiang	Alpine plateau to subtropical	Angiosperm (*Alnus*, *Cyclobalanopsis*)	Liu et al. 2021a; present study
***C.subungulatus* B.K. Cui & Shun Liu**	**East Asia (China)**	**Yunnan**	**Subtropical**	**Angiosperm (undetermined) and gymnosperm (*Pinus*)**	**Present study**
*C.subviridis* (Ryvarden & Guzmán) B.K. Cui & Shun Liu	Europe (Finland), North America (Mexico, USA)		Temperate to boreal	Gymnosperm (*Abies*, *Picea*, *Pinus*)	Miettinen et al. 2018
***C.tenuicontextus* B.K. Cui & Shun Liu**	**East Asia (China)**	**Yunnan**	**Subtropical**	**Angiosperm (undetermined) and gymnosperm (*Pinus*)**	**Present study**
*C.tenuis* B.K. Cui, Shun Liu & Y.C. Dai	East Asia (China)	Sichuan	Subtropical monsoon to Alpine plateau	Gymnosperm (*Picea*)	Liu et al. 2021a
*C.tricolor* B.K. Cui, L.L. Shen & Y.C. Dai	East Asia (China)	Sichuan, Xizang	Alpine plateau	Gymnosperm (*Abies*, *Picea*)	Shen et al. 2019
*C.ungulatus* B.K. Cui, L.L. Shen & Y.C. Dai	East Asia (China)	Sichuan	Subtropical monsoon to Alpine plateau	Angiosperm (*Castanopsis*) and gymnosperm (*Abies*)	Shen et al. 2019
*C.yanae* (Miettinen & Kotir.) B.K. Cui & Shun Liu	Europe (Russia)		Temperate continental climate	Gymnosperm (*Larix*, *Pinus*)	Miettinen et al. 2018

## ﻿Materials and methods

### ﻿Morphological studies

The examined specimens were deposited in the herbarium of the Institute of microbiology, Beijing Forestry University (BJFC), and some duplicates were deposited at the Institute of Applied Ecology, Chinese Academy of Sciences, China (IFP) and Southwest Forestry University (SWFC). Macro-morphological descriptions were based on the field notes and measurements of herbarium specimens. Special colour terms followed Petersen (1996). Micro-morphological data were obtained from the dried specimens and observed under a light microscope following Cui et al. (2019) and Liu et al. (2021b). Sections were studied at a magnification up to × 1000 using a Nikon Eclipse 80i microscope and phase contrast illumination (Nikon, Tokyo, Japan). Drawings were made with the aid of a drawing tube. Microscopic features, measurements and drawings were made from slide preparations stained with Cotton Blue and Melzer’s reagent. Spores were measured from sections cut from the tubes. To present variation in the size of basidiospores, 5% of measurements were excluded from each end of the range and extreme values are given in parentheses.

In the text the following abbreviations were used: IKI = Melzer’s reagent, IKI– = neither amyloid nor dextrinoid, KOH = 5% potassium hydroxide, CB = Cotton Blue, CB + = cyanophilous, CB – = acyanophilous, L = mean spore length (arithmetic average of all spores), W = mean spore width (arithmetic average of all spores), Q = variation in the L/W ratios between the specimens studied, n (a/b) = number of spores (a) measured from given number (b) of specimens.

### ﻿Molecular studies and phylogenetic analysis

A cetyl trimethylammonium bromide (CTAB) rapid plant genome extraction kit-DN14 (Aidlab Biotechnologies Co., Ltd, Beijing, China) was used to extract total genomic DNA from dried specimens, and performed the polymerase chain reaction (PCR) according to the manufacturer’s instructions with some modiﬁcations as described by Shen et al. (2019) and Liu et al. (2021a). The ITS regions were amplified with primer pairs ITS5 and ITS4 (White et al. 1990). The nLSU regions were amplified with primer pairs LR0R and LR7 (http://www.biology.duke.edu/fungi/mycolab/primers.htm). The nSSU regions were amplified with primer pairs NS1 and NS4 (White et al. 1990). The mtSSU regions were amplified with primer pairs MS1 and MS2 (White et al. 1990). RPB1 was ampliﬁed with primer pairs RPB1-Af and RPB1-Cr (Matheny et al. 2002). RPB2 was amplified with primer pairs fRPB2-f5F and bRPB2-7.1R (Matheny 2005). Part of TEF was amplified with primer pairs EF1-983 F and EF1-1567R (Rehner 2001).

The PCR cycling schedule for ITS, mtSSU and TEF included an initial denaturation at 95 °C for 3 min, followed by 35 cycles at 94 °C for 40 s, 54 °C for ITS and mtSSU, 54–55 °C for TEF for 45 s, 72 °C for 1 min, and a final extension at 72 °C for 10 min. The PCR cycling schedule for nLSU and nSSU included an initial denaturation at 94 °C for 1 min, followed by 35 cycles at 94 °C for 30 s, 50 °C for nLSU and 52 °C for nSSU for 1 min, 72 °C for 1.5 min, and a final extension at 72 °C for 10 min. The PCR procedure for RPB1 and RPB2 follow Justo and Hibbett (2011) with slight modiﬁcations: initial denaturation at 94 °C for 2 min, followed by 10 cycles at 94 °C for 40 s, 60 °C for 40 s and 72 °C for 2 min, then followed by 37 cycles at 94 °C for 45 s, 55 °C for 1.5 min and 72 °C for 2 min, and a ﬁnal extension of 72 °C for 10 min. The PCR products were purified and sequenced at Beijing Genomics Institute (BGI), China, with the same primers. All newly generated sequences were deposited at GenBank (Table 1).

Additional sequences were downloaded from GenBank (Table 1). All sequences of ITS, nLSU, nSSU, mtSSU, RPB1, RPB2 and TEF were respectively aligned in MAFFT 7 (Katoh and Standley 2013; http://mafft.cbrc.jp/alignment/server/) and manually adjusted in BioEdit (Hall 1999). Alignments were spliced in Mesquite (Maddison and Maddison 2017). The missing sequences were coded as ‘‘N’’. Ambiguous nucleotides were coded as ‘‘N’’. The ﬁnal concatenated sequence alignment was deposited at TreeBase (http://purl.org/phylo/treebase; submission ID: 29010).

Most parsimonious phylogenies were inferred from the combined 2-gene dataset (ITS+TEF) and 7-gene dataset (ITS+nLSU+nSSU+mtSSU+RPB1+RPB2+TEF), and their congruences were evaluated with the incongruence length difference (ILD) test (Farris et al. 1994) implemented in PAUP* 4.0b10 (Swofford 2002), under heuristic search and 1000 homogeneity replicates. Phylogenetic analyses approaches followed Liu et al. (2019) and Sun et al. (2020). In phylogenetic reconstruction, the sequences of *Antrodiaserpens* (Fr.) Donk and *A.tanakae* (Murrill) Spirin & Miettinen obtained from GenBank were used as outgroups. Maximum parsimony analysis was applied to the combined multiple genes datasets, and the tree construction procedure was performed in PAUP* version 4.0b10. All characters were equally weighted and gaps were treated as missing data. Trees were inferred using the heuristic search option with TBR branch swapping and 1000 random sequence additions. Max-trees were set to 5000, branches of zero length were collapsed and all parsimonious trees were saved. Clade robustness was assessed using a bootstrap (BT) analysis with 1000 replicates (Felsenstein 1985). Descriptive tree statistics tree length (TL), consistency index (CI), retention index (RI), rescaled consistency index (RC), and homoplasy index (HI) were calculated for each most Parsimonious Tree (MPT) generated. RAxmL v.7.2.8 was used to construct a maximum likelihood (ML) tree with a GTR+G+I model of site substitution including estimation of Gamma-distributed rate heterogeneity and a proportion of invariant sites (Stamatakis 2006). The branch support was evaluated with a bootstrapping method of 1000 replicates (Hillis and Bull 1993). The phylogenetic tree was visualized using FigTree v1.4.2 (http://tree.bio.ed.ac.uk/software/figtree/).

MrModeltest 2.3 (Posada and Crandall 1998; Nylander 2004) was used to determine the best-fit evolution model for the combined multi-gene dataset for Bayesian inference (BI). Bayesian inference was calculated with MrBayes 3.1.2 with a general time reversible (GTR) model of DNA substitution and a gamma distribution rate variation across sites (Ronquist and Huelsenbeck 2003). Four Markov chains were run for 2 runs from random starting trees for 1.8 million generations (ITS+TEF), for 3.5 million generations (ITS+nLSU+nSSU+mtSSU+RPB1+RPB2+TEF) and trees were sampled every100 generations. The first one-fourth generations were discarded as burn-in. A majority rule consensus tree of all remaining trees was calculated. Branches that received bootstrap support for maximum parsimony (MP), maximum likelihood (ML) and Bayesian posterior probabilities (BPP) greater than or equal to 75% (MP and ML) and 0.95 (BPP) were considered as significantly supported, respectively.

## ﻿Results

### ﻿Phylogeny

**Figure 2.**
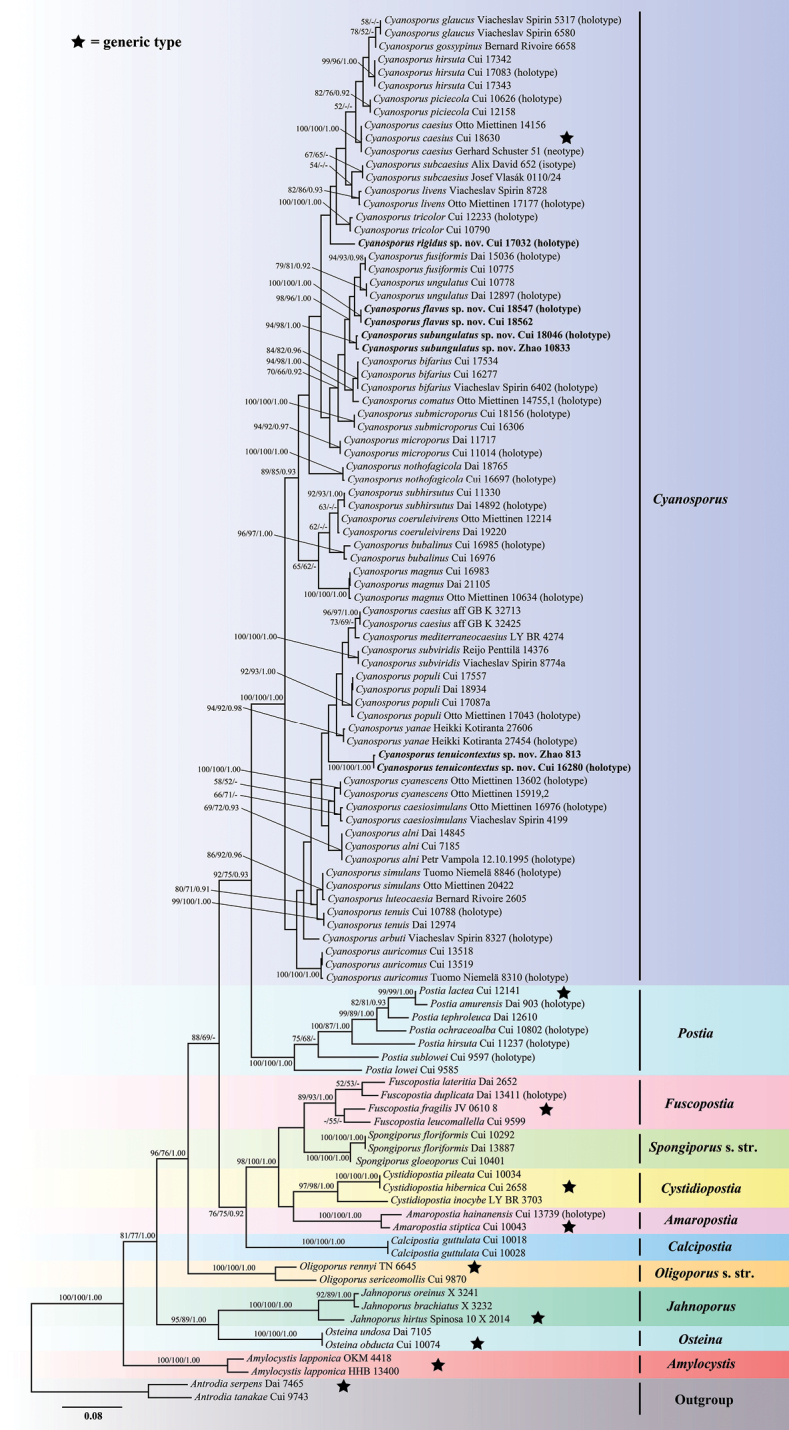
Maximum likelihood tree illustrating the phylogeny of *Cyanosporus* and its related genera in the antrodia clade based on the combined sequences dataset of ITS+TEF. Branches are labelled with maximum likelihood bootstrap higher than 50%, parsimony bootstrap proportions higher than 50% and Bayesian posterior probabilities more than 0.90 respectively. Bold names = New species.

The combined 2-gene (ITS+TEF) sequences dataset had an aligned length of 1015 characters, of which 502 characters were constant, 62 were variable and parsimony-uninformative, and 451 were parsimony-informative. MP analysis yielded 10 equally parsimonious trees (TL = 2396, CI = 0.379, RI = 0.735, RC = 0.279, HI = 0.621). The best model for the concatenate sequence dataset estimated and applied in the Bayesian inference was GTR+I+G with equal frequency of nucleotides. ML analysis resulted in a similar topology as MP and Bayesian analyses, and only the ML topology is shown in Fig. 2.

**Figure 3.**
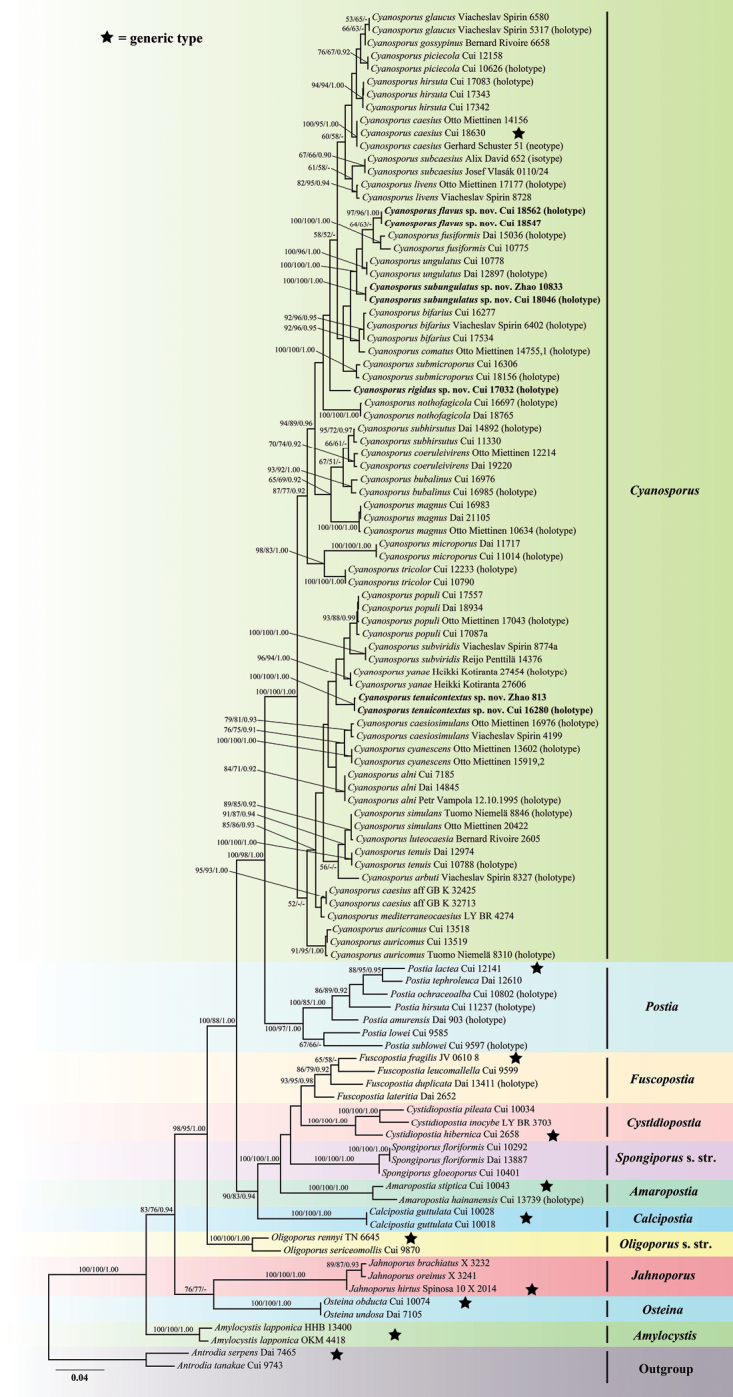
Maximum likelihood tree illustrating the phylogeny of *Cyanosporus* and its related genera in the antrodia clade based on the combined sequences dataset of ITS+nLSU+nSSU+mtSSU+RPB1+RPB2+TEF. Branches are labelled with maximum likelihood bootstrap higher than 50%, parsimony bootstrap propwortions higher than 50% and Bayesian posterior probabilities more than 0.90 respectively. Bold names = New species.

The combined 7-gene (ITS+nLSU+nSSU+mtSSU+RPB1+RPB2+TEF) sequences dataset had an aligned length of 5634 characters, of which 3843 characters were constant, 247 were variable and parsimony-uninformative, and 1544 were parsimony-informative. MP analysis yielded 23 equally parsimonious trees (TL = 5756, CI = 0.468, RI = 0.752, RC = 0.352, HI = 0.532). The best model for the concatenate sequence dataset estimated and applied in the Bayesian inference was GTR+I+G with equal frequency of nucleotides. ML analysis resulted in a similar topology as MP and Bayesian analyses, and only the ML topology is shown in Fig. 3.

The phylogenetic trees inferred from ITS+TEF and ITS+nLSU+nSSU+mtSSU+RPB1+RPB2+TEF gene sequences were all obtained from 106 fungal samples representing 65 taxa of *Cyanosporus* and its related genera within the antrodia clade. 74 samples representing 35 taxa of *Cyanosporus* clustered together and separated from species of *Postia* and other related genera. As for *Cyanosporus*, the sequences used in phylogenetic analyses include 28 holotype specimen sequences, one isotype specimen sequence and one neotype specimen sequence (Table 1).

### ﻿Taxonomy

#### ﻿ Cyanosporus flavus

Taxon classificationFungiPolyporalesPolyporaceae

B.K. Cui & Shun Liu sp. nov.

CC2A2AEE-34B1-5398-A726-C5105333B75F

842319

Figs 4 , 5

##### Diagnosis.

*Cyanosporusflavus* is characterised by flabelliform to semicircular and hirsute pileus with ash grey to light vinaceous grey pileal surface when fresh, buff to lemon-chrome pore surface when dry, and allantoid and slightly curved basidiospores (4.6–5.2 × 0.8–1.3 μm).

##### Holotype.

China. Sichuan Province, Jiuzhaigou County, on stump of *Picea* sp., 19.IX.2020, Cui 18547 (BJFC 035408).

##### Etymology.

*Flavus* (Lat.): referring to its lemon-chrome pore surface when dry.

##### Fruiting body.

Basidiomata annual, pileate, soft and watery, without odour or taste when fresh, becoming corky to fragile and light in weight upon drying. Pileus flabelliform to semicircular, projecting up to 3.2 cm, 5.7 cm wide and 0.9 cm thick at base. Pileal surface ash-grey to light vinaceous grey when fresh, becoming pale mouse-grey to mouse-grey when dry, hirsute; margin acute to slightly obtuse, white with a little blue tint when fresh, olivaceous buff to greyish brown when dry. Pore surface white to cream when fresh, becoming buff to lemon-chrome when dry; sterile margin narrow to almost lacking; pores angular, 5–7 per mm; dissepiments thin, entire to lacerate. Context white to cream, soft corky, up to 6 mm thick. Tubes pale mouse-grey to ash-grey, fragile, up to 4 mm long.

##### Hyphal structure.

Hyphal system monomitic; generative hyphae with clamp connections, IKI–, CB–; hyphae unchanged in KOH.

##### Context.

Generative hyphae hyaline, thin- to slightly thick-walled with a wide lumen, occasionally branched, loosely interwoven, 2.7–6.5 μm in diam.

##### Tubes.

Generative hyphae hyaline, thin- to slightly thick-walled with a wide lumen, rarely branched, interwoven, 2.2–4.7 μm in diam. Cystidia absent; cystidioles present, fusoid, thin-walled, 12.3–17.8 × 2.2–3.5 μm. Basidia clavate, bearing four sterigmata and a basal clamp connection, 13.2–16.5 × 3.2–5.5 μm; basidioles dominant, in shape similar to basidia, but smaller, 12.6–15.7 × 2.9–5.2 μm.

**Figure 4.**
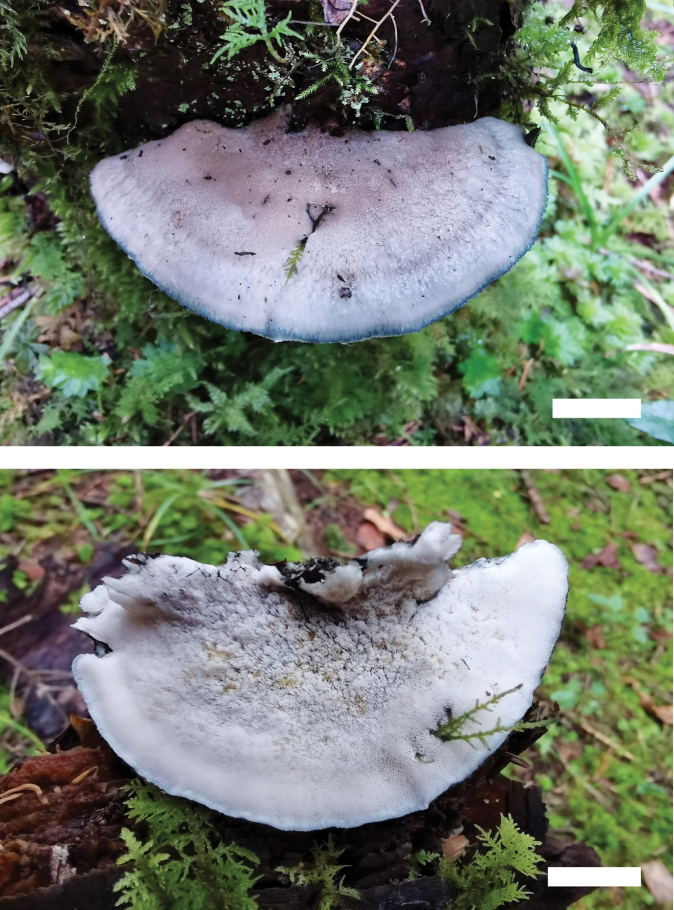
Basidiomata of *Cyanosporusflavus* (Holotype, Cui 18547). Scale bar: 1 cm. The upper figure is the upper surface and the lower figure is the lower surface of the basidiomata.

**Figure 5.**
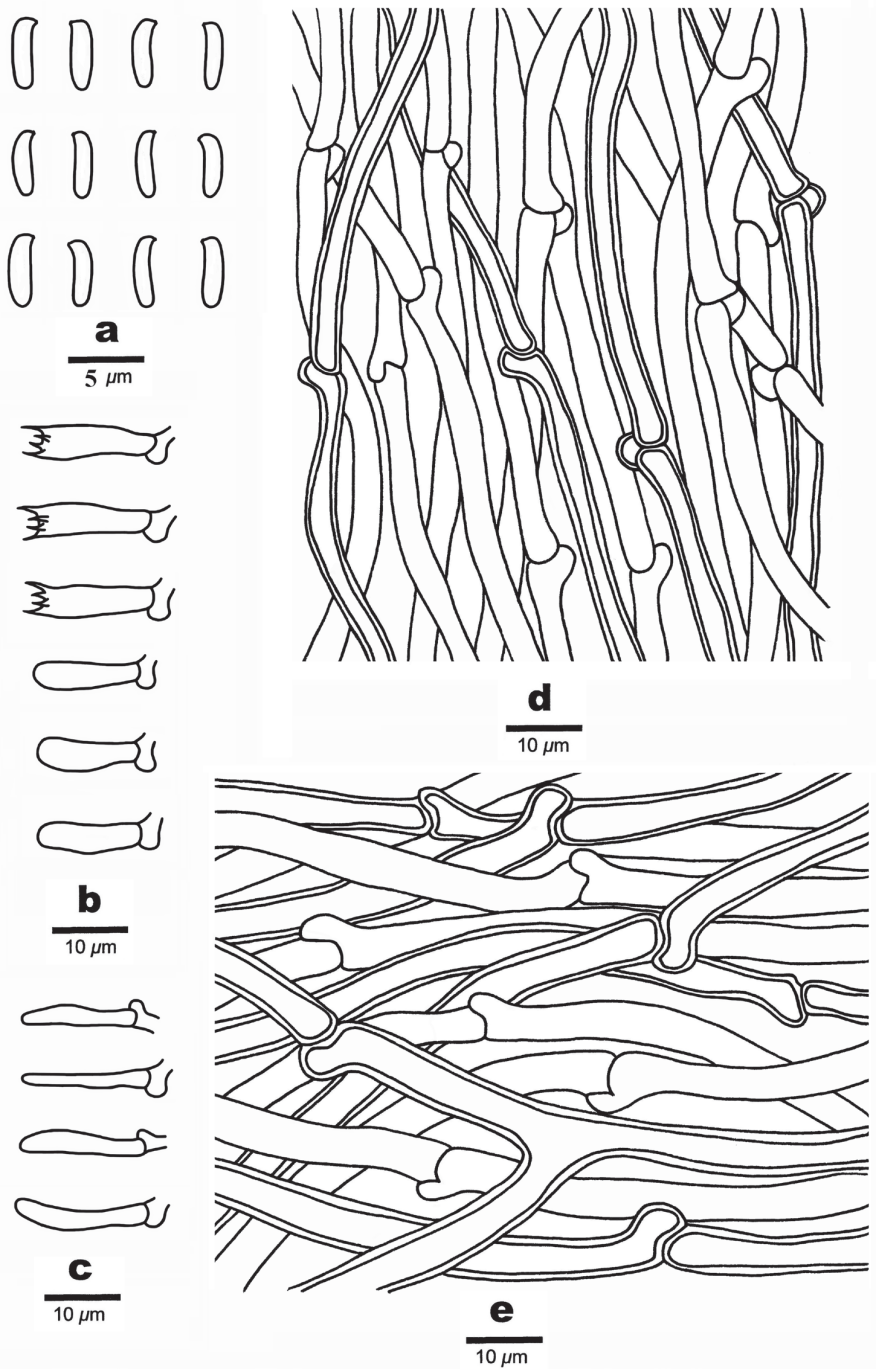
Microscopic structures of *Cyanosporusflavus* (Holotype, Cui 18547) **a** basidiospores **b** basidia and basidioles **c** cystidioles **d** hyphae from trama **e** hyphae from context. Drawings by: Shun Liu.

##### Spores.

Basidiospores slim allantoid, slightly curved, hyaline, thin- to slightly thick-walled, smooth, IKI–, CB–, 4.6–5.2 × 0.8–1.3 μm, L = 5 μm, W = 0.99 μm, Q = 4.96–5.25 (n = 60/2).

##### Type of rot.

Brown rot.

##### Additional specimen (paratype) examined.

China. Sichuan Province, Jiuzhaigou County, Jiuzhaigou Nature Reserve, on fallen trunk of *Abies* sp., 20.IX.2020, Cui 18562 (BJFC 035423).

#### ﻿ Cyanosporus rigidus

Taxon classificationFungiPolyporalesPolyporaceae

B.K. Cui & Shun Liu sp. nov.

C5D8B882-5106-5A28-BA24-A125D6A7D9C1

842320

Figs 6 , 7

##### Diagnosis.

*Cyanosporusrigidus* is characterised by corky, hard corky to rigid basidiomata with a buff yellow to clay-buff and tomentose pileal surface when fresh, becoming olivaceous buff to greyish brown when dry, smaller and cylindrical to allantoid basidiospores (3.7–4.2 × 0.9–1.3 μm).

##### Holotype.

China. Yunnan Province, Yulong County, Laojun Mountain, Jiushijiu Longtan, on fallen trunk of *Abies* sp., 15.IX.2018, Cui 17032 (BJFC 030331).

##### Etymology.

*Rigidus* (Lat.): referring to the rigid basidiomata.

##### Fruiting body.

Basidiomata annual, pileate, corky, without odour or taste when fresh, becoming hard corky to rigid upon drying. Pileus flabelliform, projecting up to 1.6 cm, 3.8 cm wide and 0.6 cm thick at base. Pileal surface tomentose, buff yellow to clay-buff, when fresh, becoming smooth, rugose, olivaceous buff to greyish brown when dry; margin obtuse. Pore surface white to cream when fresh, becoming buff-yellow to pinkish buff when dry; sterile margin narrow to almost lacking; pores angular, 5–8 per mm; dissepiments thin, entire to lacerate. Context cream to buff, hard corky, up to 4 mm thick. Tubes cream to pinkish buff, brittle, up to 5 mm long.

**Figure 6.**
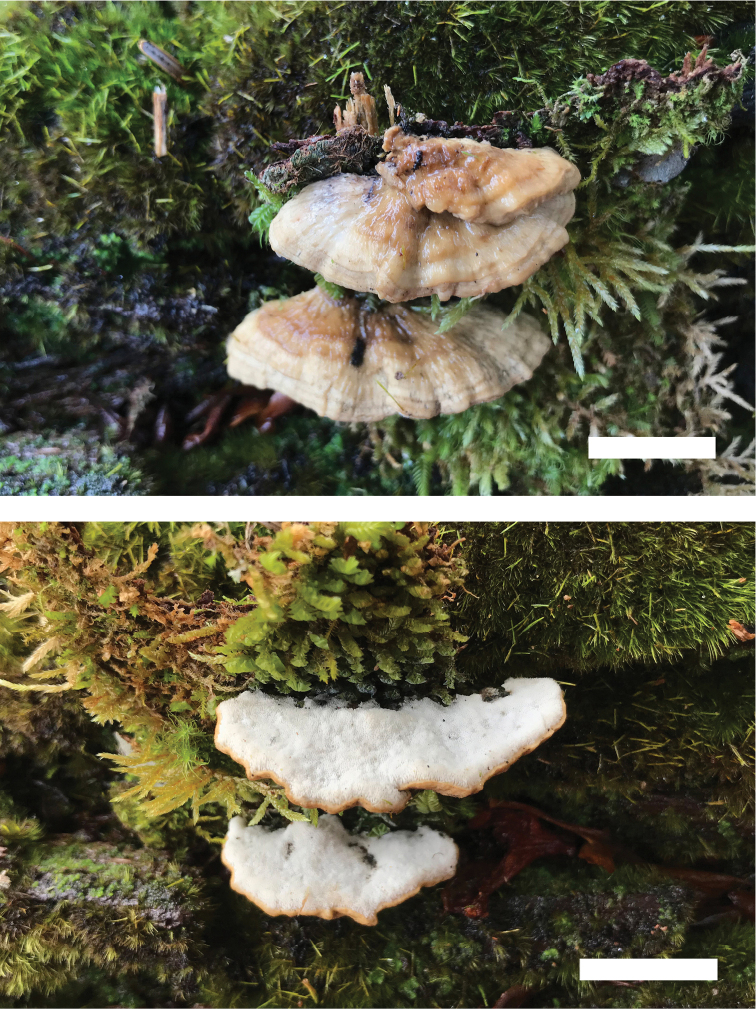
Basidiomata of *Cyanosporusrigidus* (Holotype, Cui 17032). Scale bar: 1.5 cm. The upper figure is the upper surface and the lower figure is the lower surface of the basidiomata.

**Figure 7.**
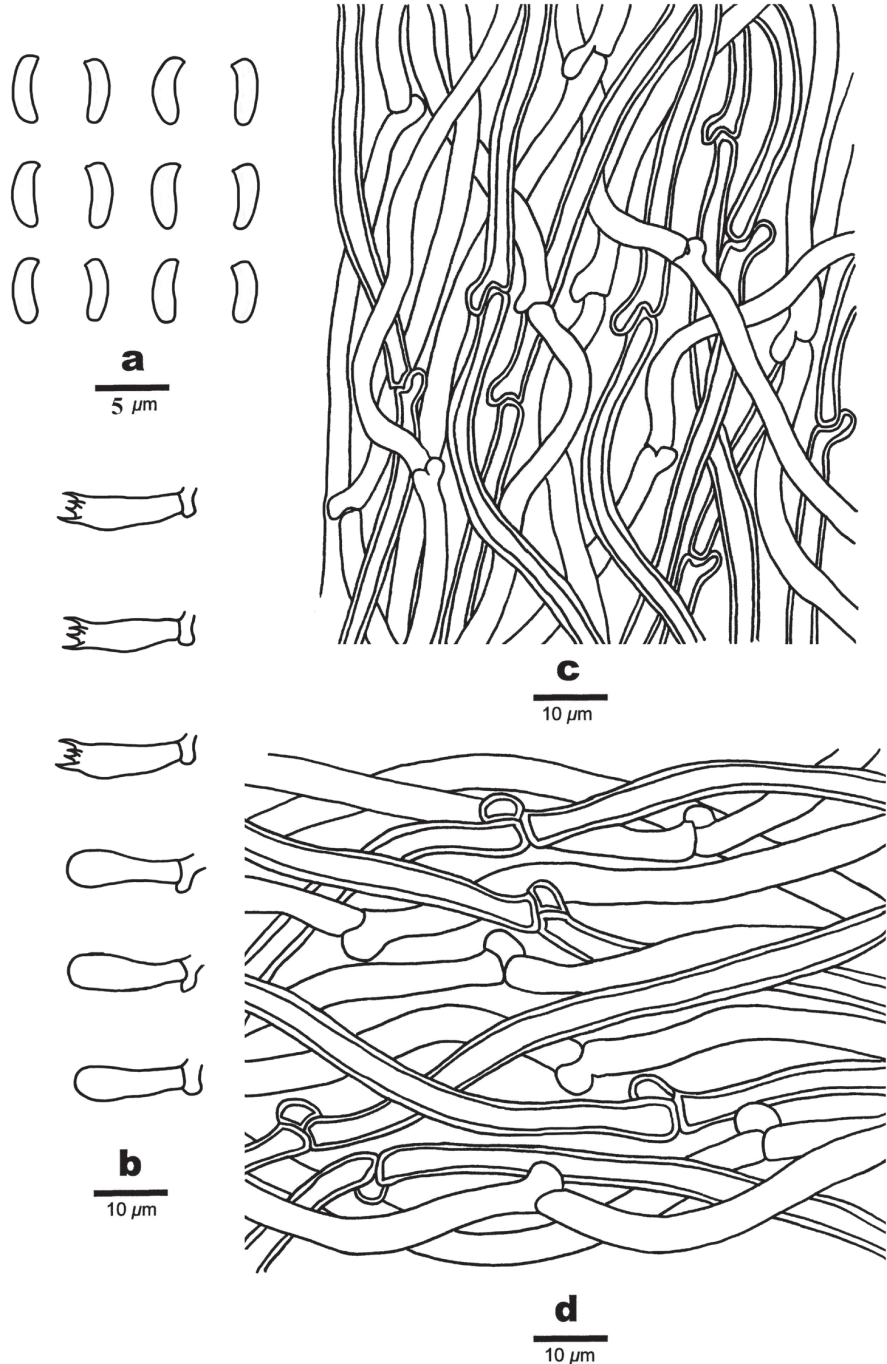
Microscopic structures of *Cyanosporusrigidus* (Holotype, Cui 17032) **a** basidiospores **b** basidia and basidioles **c** hyphae from trama **d** hyphae from context. Drawings by: Shun Liu.

##### Hyphal structure.

Hyphal system monomitic; generative hyphae with clamp connections, IKI–, CB–; hyphae unchanged in KOH.

##### Context.

Generative hyphae hyaline, thin- to slightly thick-walled with a wide lumen, rarely branched, loosely interwoven, 2.2–5 μm in diam.

##### Tubes.

Generative hyphae hyaline, thin- to slightly thick-walled with a wide lumen, occasionally branched, interwoven, 2–4 μm in diam. Cystidia and cystidioles absent. Basidia clavate, bearing four sterigmata and a basal clamp connection, 12.4–14.8 × 3–4.2 μm; basidioles dominant, in shape similar to basidia, but smaller, 11.8–13.9 × 2.6–4 μm.

##### Spores.

Basidiospores allantoid to cylindrical, slightly curved, hyaline, thin- to slightly thick-walled, smooth, IKI–, CB–, (3.5–)3.7–4.2 × (0.8–)0.9–1.3(–1.4) μm, L = 3.94 μm, W = 1.09 μm, Q = 3.66 (n = 60/1).

##### Type of rot.

Brown rot.

#### ﻿ Cyanosporus subungulatus

Taxon classificationFungiPolyporalesPolyporaceae

B.K. Cui & Shun Liu sp. nov.

803D7DD9-C55D-568E-9E1A-5F69573F850D

842321

Figs 8 , 9

##### Diagnosis.

*Cyanosporussubungulatus* is characterised by shell-shaped pileus with a pale mouse-grey to ash-grey pileal surface when fresh, dark-grey to mouse-grey when dry, allantoid to cylindrical and slightly curved basidiospores (4.5–5.2 × 1.1–1.4 μm).

##### Holotype.

China. Yunnan Province, Yangbi County, Shimenguan Nature Reserve, on fallen trunk of *Pinus* sp., 6.IX.2019, Cui 18046 (BJFC 034905).

##### Etymology.

*Subungulatus* (Lat.): referring to the species resembling *Cyanosporusungulatus* in morphology.

##### Fruiting body.

Basidiomata annual, pileate, soft corky, without odour or taste when fresh, becoming corky to fragile and light in weight upon drying. Pileus shell-shaped, projecting up to 1.7 cm, 2.8 cm wide and 1.2 cm thick at base. Pileal surface velutinate, pale mouse-grey to ash-grey when fresh, becoming smooth, rugose, dark-grey to mouse-grey when dry; margin obtuse. Pore surface white to cream when fresh, becoming cream to pinkish buff when dry; sterile margin narrow to almost lacking; pores round, 4–6 per mm; dissepiments thin, entire to lacerate. Context white to cream, soft corky, up to 5 mm thick. Tubes pale mouse-grey to ash-grey, fragile, up to 6 mm long.

**Figure 8.**
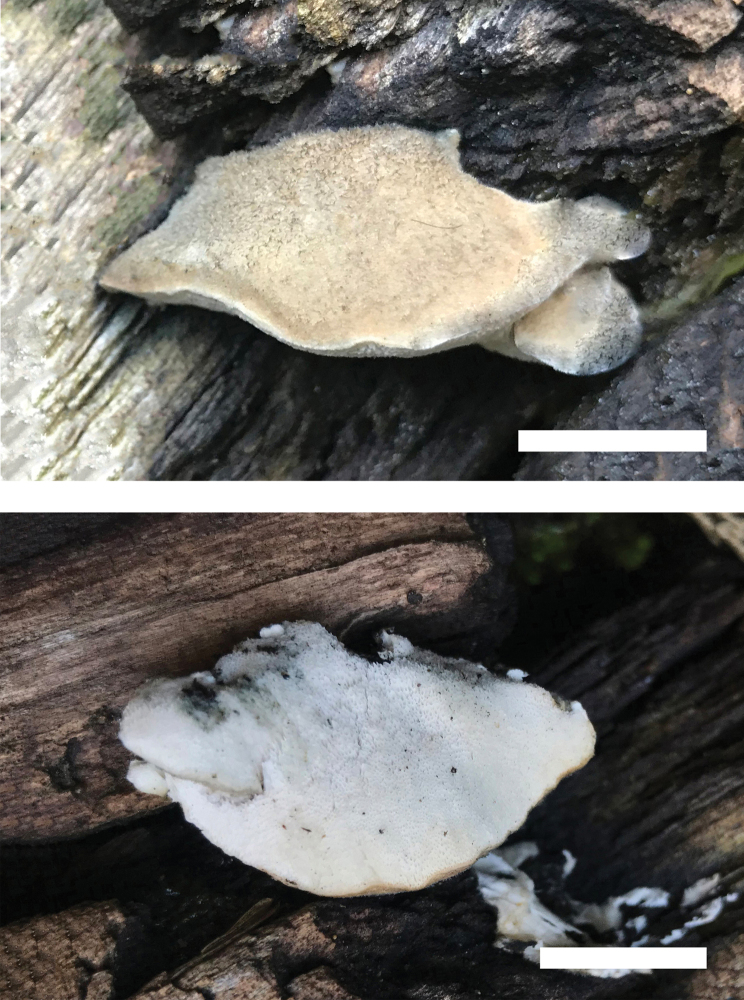
Basidiomata of *Cyanosporussubungulatus* (Holotype, Cui 18046). Scale bar: 10 mm. The upper figure is the upper surface and the lower figure is the lower surface of the basidiomata.

**Figure 9.**
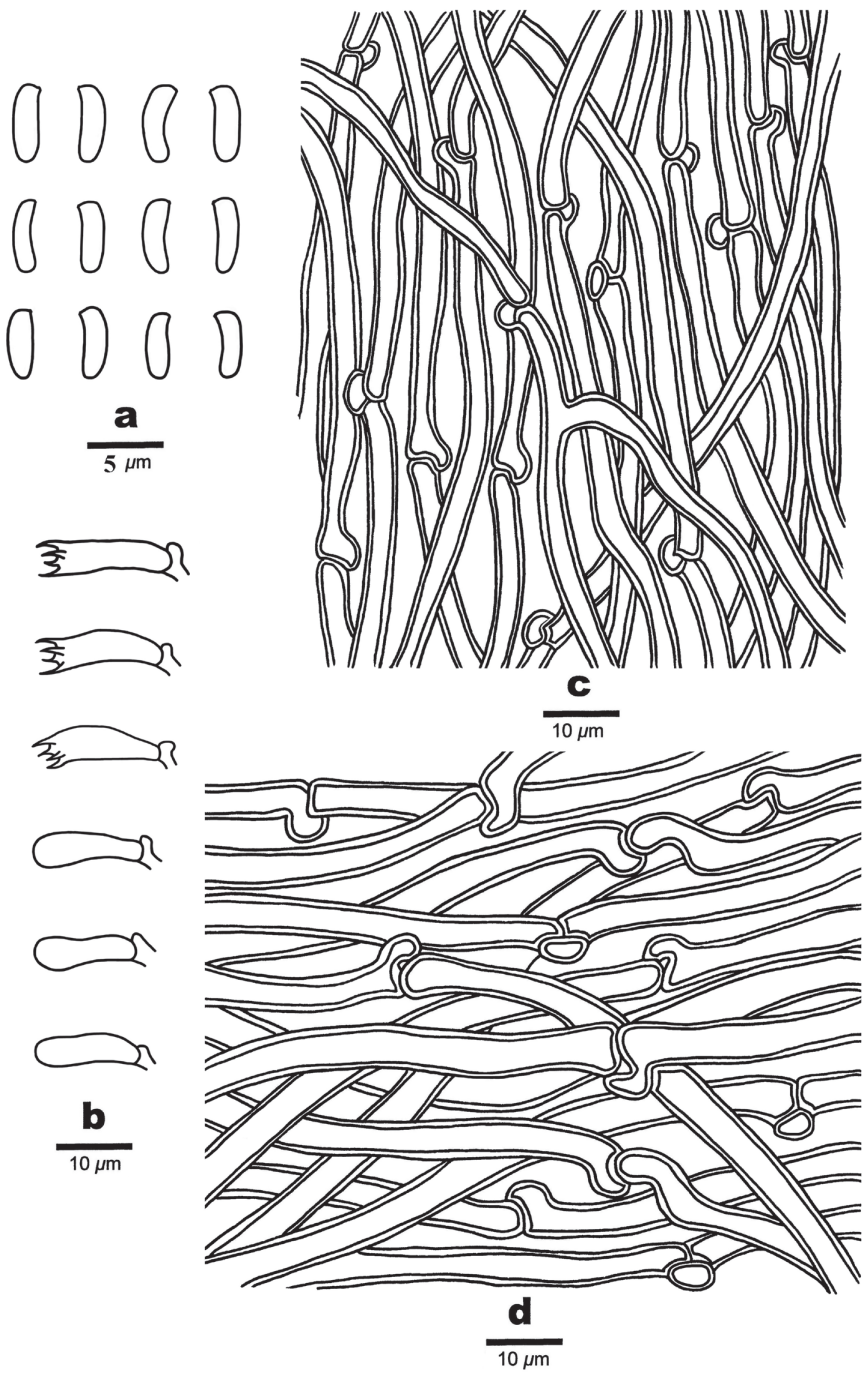
Microscopic structures of *Cyanosporussubungulatus* (Holotype, Cui 18046) **a** basidiospores **b** basidia and basidioles **c** hyphae from trama **d** hyphae from context. Drawings by: Shun Liu.

##### Hyphal structure.

Hyphal system monomitic; generative hyphae with clamp connections, IKI–, CB–; hyphae unchanged in KOH.

##### Context.

Generative hyphae hyaline, slightly thick-walled with a wide lumen, rarely branched, loosely interwoven, 2.5–6.4 μm in diam.

##### Tubes.

Generative hyphae hyaline, slightly thick-walled with a wide lumen, occasionally branched, interwoven, 2–4.2 μm in diam. Cystidia and cystidioles absent. Basidia clavate, bearing four sterigmata and a basal clamp connection, 13.6–17.8 × 3–5.5 μm; basidioles dominant, in shape similar to basidia, but smaller, 12.8–17.2 × 2.4–5.2 μm.

##### Spores.

Basidiospores allantoid to cylindrical, slightly curved, hyaline, thin- to slightly thick-walled, smooth, IKI–, CB–, (4.3–)4.5–5.2 × 1.1–1.4 μm, L = 4.73 μm, W = 1.22 μm, Q = 3.48–3.66 (n = 60/2).

##### Type of rot.

Brown rot.

##### Additional specimen (paratype) examined.

China, Yunnan Province, Xichou County, Xiaoqiaogou Nature Reserve, on fallen angiosperm trunk, 14.I.2019, Zhao 10833 (SWFC 010833).

#### ﻿ Cyanosporus tenuicontextus

Taxon classificationFungiPolyporalesPolyporaceae

B.K. Cui & Shun Liu sp. nov.

6A2E6771-2883-597D-9041-F2C47FA6B3E9

842323

Figs 10 , 11

##### Diagnosis.

*Cyanosporustenuicontextus* is characterised by flabelliform pileus with a velutinate, cream to pinkish buff with a little blue tint pileal surface when fresh, becoming glabrous, light vinaceous grey to pale mouse-grey when dry, small and round pores (6–8 per mm), thin context (up to 0.8 mm) and allantoid basidiospores (3.8–4.3 × 0.8–1.2 μm).

##### Holotype.

China. Yunnan Province, Lanping County, Tongdian Town, Luoguqing, on fallen trunk of *Pinus* sp., 19.IX.2017, Cui 16280 (BJFC 029579).

##### Etymology.

*Tenuicontextus* (Lat.): referring to the species having thin context.

##### Fruiting body.

Basidiomata annual, pileate, soft corky, without odour or taste when fresh, becoming corky to fragile and light in weight upon drying. Pileus flabelliform, projecting up to 1.3 cm, 3.2 cm wide and 0.5 cm thick at base. Pileal surface velutinate, cream to pinkish buff with a little blue tint when fresh, becoming glabrous, light vinaceous grey to pale mouse-grey when dry; margin acute. Pore surface white to cream when fresh, becoming pinkish buff to buff when dry; sterile margin narrow to almost lacking; pores round, 6–8 per mm; dissepiments thin, entire to lacerate. Context cream to buff, soft corky, up to 0.8 mm thick. Tubes pale mouse-grey to buff, fragile, up to 4.3 mm long.

**Figure 10.**
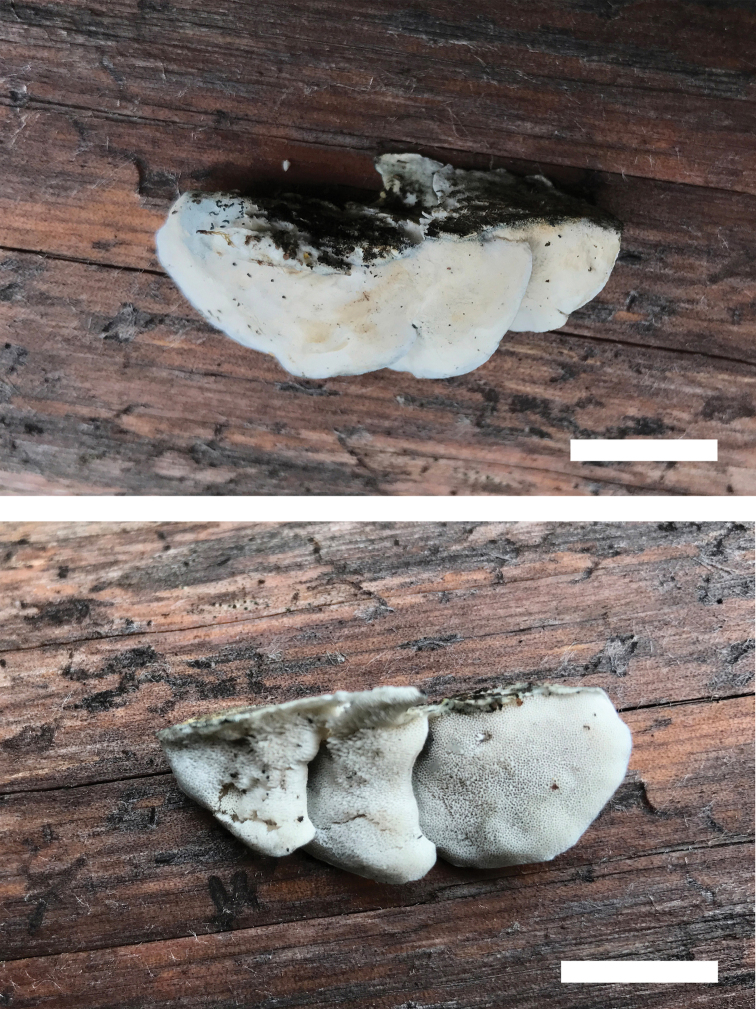
Basidiomata of *Cyanosporustenuicontextus* (Holotype, Cui 16280). Scale bar: 1 cm. The upper figure is the upper surface and the lower figure is the lower surface of the basidiomata.

**Figure 11.**
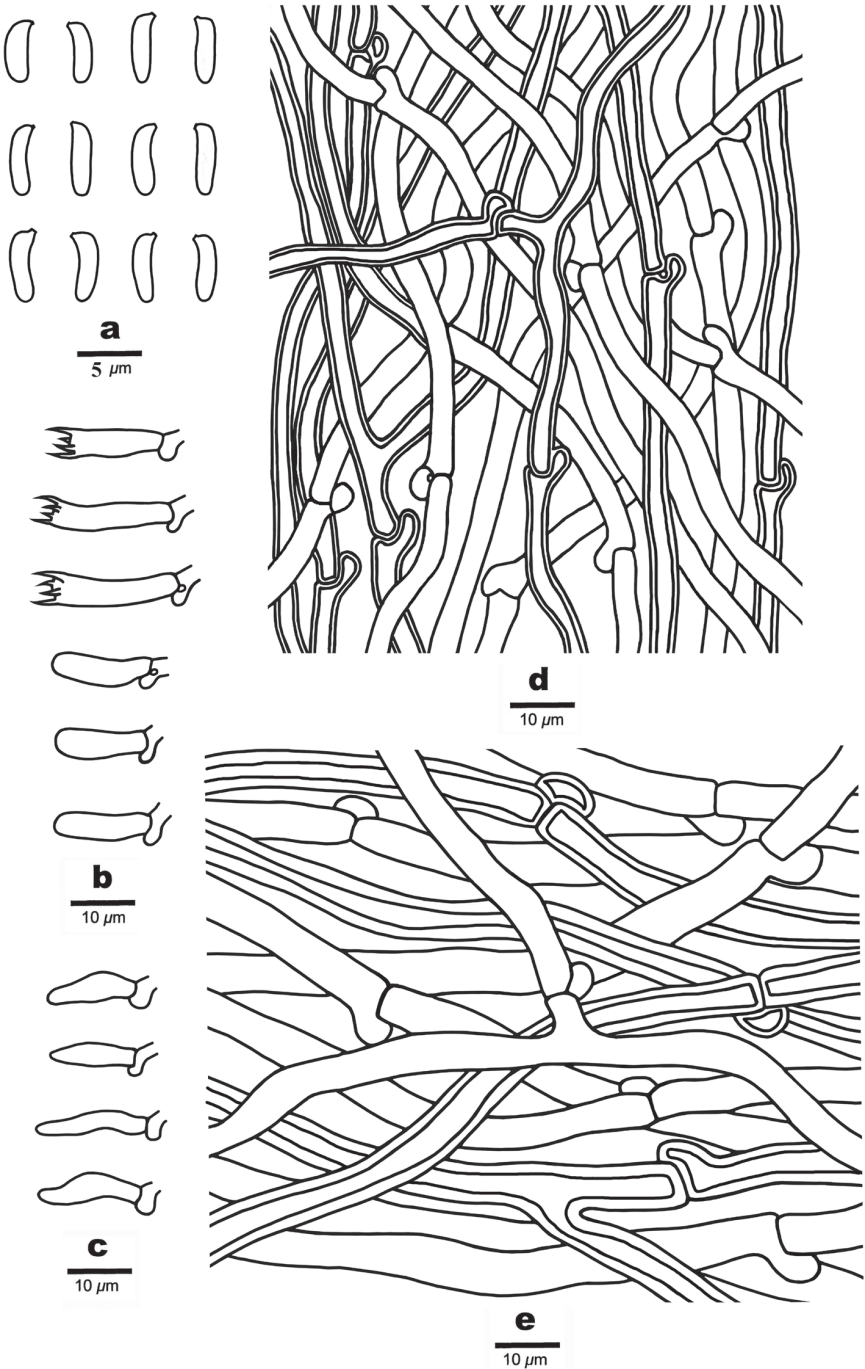
Microscopic structures of *Cyanosporustenuicontextus* (Holotype, Cui 16280) **a** basidiospores **b** basidia and basidioles. **c** cystidioles **d** hyphae from trama **e** hyphae from context. Drawings by: Shun Liu.

##### Hyphal structure.

Hyphal system monomitic; generative hyphae with clamp connections, IKI–, CB–; hyphae unchanged in KOH.

##### Context.

Generative hyphae hyaline, thin- to slightly thick-walled with a wide lumen, occasionally branched, loosely interwoven, 2.3–5.5 μm in diam.

##### Tubes.

Generative hyphae hyaline, thin- to slightly thick-walled with a wide lumen, occasionally branched, interwoven, 2–4 μm in diam. Cystidia absent; cystidioles present, fusoid, thin-walled, 9.5–14.6 × 2.8–3.4 μm. Basidia clavate, bearing four sterigmata and a basal clamp connection, 11.7–16.8 × 3.4–4.3 μm; basidioles dominant, in shape similar to basidia, but smaller, 10.6–14.7 × 2.9–3.6 μm.

##### Spores.

Basidiospores allantoid, slightly curved, hyaline, thin- to slightly thick-walled, smooth, IKI–, CB–, (3.7–)3.8–4.3 × 0.8–1.2 μm, L = 3.97 μm, W = 1.02 μm, Q = 3.78–4.26 (n = 60/2).

##### Type of rot.

Brown rot.

##### Additional specimen (paratype) examined.

China. Yunnan Province, Yuxi, Xinping County, Mopanshan National Forest Park, on angiosperm stump, 16.I.2017, Zhao 813 (SWFC 000813).

## ﻿Discussion

In the current phylogenetic analyses based on the combined datasets of ITS+TEF and ITS+nLSU+mtSSU+nSSU+RPB1+RPB2+TEF sequences, species of *Cyanosporus* formed a highly supported lineage, distant from *Postia* and other brown-rot fungal genera (Figs 2, 3) and consistent with previous studies (Shen et al. 2019; Liu et al. 2021a). Based on morphological characters and phylogenetic analyses, 35 species are accepted in *Cyanosporus* around the world, including four new species from China, viz., *C.flavus*, *C.rigidus*, *C.subungulatus* and *C.tenuicontextus*. The main ecological habits of the species in *Cyanosporus* with an emphasis on distribution areas and host trees are provided in Table 2.

**Table 2.** A list of species, specimens, and GenBank accession number of sequences used for phylogenetic analyses in this study.

Species	Sample no.	Locality	GenBank accessions							References
			ITS	nLSU	mtSSU	nSSU	RPB1	RPB2	TEF	
*Amaropostiahainanensis*	Cui 13739 (holotype)	China	KX900909	KX900979	KX901053	KX901123	KX901171	KX901223		Shen et al. 2019
*A.stiptica*	Cui 10043	China	KX900906	KX900976	KX901046	KX901119	KX901167	KX901219		Shen et al. 2019
*Amylocystislapponica*	HHB-13400	United States	KC585237	KC585059						Ortiz-Santana et al. 2013
*A.lapponica*	OKM-4418	United States	KC585238	KC585060						Ortiz-Santana et al. 2013
*Antrodiaserpens*	Dai 7465	Luxembourg	KR605813	KR605752	KR606013	KR605913		KR610832	KR610742	Han et al. 2016
*A.tanakae*	Cui 9743	China	KR605814	KR605753	KR606014	KR605914		KR610833	KR610743	Han et al. 2016
*Calcipostiaguttulata*	Cui 10018	China	KF727432	KJ684978	KX901065	KX901138	KX901181	KX901236	KX901276	Shen et al. 2019
*C.guttulata*	Cui 10028	China	KF727433	KJ684979	KX901066	KX901139	KX901182	KX901237	KX901277	Shen et al. 2019
*Cyanosporusalni*	Petr Vampola 12.10.1995 (holotype)	Slovakia	MG137026							Miettinen et al. 2018
*C.alni*	Cui 7185	China	KX900879	KX900949	KX901017	KX901092	KX901155	KX901202	KX901254	Shen et al. 2019
*C.alni*	Dai 14845	Poland	KX900880	KX900950	KX901018	KX901093	KX901156	KX901203	KX901255	Shen et al. 2019
*C.arbuti*	Viacheslav Spirin 8327 (holotype)	United States	MG137039						MG137132	Miettinen et al. 2018
*C.auricomus*	Cui 13518	China	KX900887	KX900957	KX901025	KX901100		KX901209		Shen et al. 2019
*C.auricomus*	Cui 13519	China	KX900888	KX900958	KX901026	KX901101				Shen et al. 2019
*C.auricomus*	Tuomo Niemelä 8310 (holotype)	Finland	MG137040							Miettinen et al. 2018
*C.bifarius*	Viacheslav Spirin 6402 (holotype)	Russia	MG137043						MG137133	Miettinen et al. 2018
*C.bifarius*	Cui 17534	China	OL423598*	OL423608*	OL437195*	OL423620*	OL444985*	OL446999*	OL444994*	Present study
*C.bifarius*	Cui 16277	China	OL423599*	OL423609*	OL437196*	OL423621*	OL444986*	OL447000*	OL444995*	Present study
*C.bubalinus*	Cui 16976	China	MW182172	MW182225	MW182208	MW182189	MW191547	MW191563	MW191530	Liu et al. 2021a
*C.bubalinus*	Cui 16985 (holotype)	China	MW182173	MW182226	MW182209	MW182190	MW191548	MW191564	MW191531	Liu et al. 2021a
*C.caesiosimulans*	Viacheslav Spirin 4199	Russia	MG137061						MG137140	Miettinen et al. 2018
*C.caesiosimulans*	Otto Miettinen 16976 (holotype)	United States	MG137054						MG137137	Miettinen et al. 2018
*C.caesius*	Gerhard Schuster 51 (neotype)	Germany	MG137045							Miettinen et al. 2018
*C.caesius*	Otto Miettinen 14156	Finland	MG137048						MG137134	Miettinen et al. 2018
*C.caesius*	Cui 18630	France	OL423600*	OL423610*	OL437197*	OL423622*			OL444996*	Present study
*C.caesius* aff GB	K 32713	United Kingdom	AY599576							Miettinen et al. 2018
*C.caesius* aff GB	K 32425	United Kingdom	AY599575							Miettinen et al. 2018
*C.coeruleivirens*	Otto Miettinen 12214	Indonesia	MG137063							Miettinen et al. 2018
*C.coeruleivirens*	Dai 19220	China	MW182174	MW182227	MW182210	MW182191	MW191549		MW191532	Liu et al. 2021a
*C.comatus*	Otto Miettinen 14755,1 (holotype)	United States	MG137066							Miettinen et al. 2018
*C.cyanescens*	Otto Miettinen 13602 (holotype)	Finland	MG137067						MG137142	Miettinen et al. 2018
*C.cyanescens*	Otto Miettinen 15919,2	Spain	MG137071						MG137144	Miettinen et al. 2018
*C.flavus*	Cui 18547	China	MW448564*	MW448561*		MW448557*	MW452596*	MW452599*	**MW452601**	**Present study**
*C.flavus*	Cui 18562 (holotype)	China	MW448565*	MW448562*		MW448558*	MW452597*	MW452600*	**MW452602**	**Present study**
*C.fusiformis*	Cui 10775	China	KX900868	KX900938	KX901006	KX901081		KX901191	KX901245	Shen et al. 2019
*C.fusiformis*	Dai 15036 (holotype)	China	KX900867	KX900937	KX901005	KX901080		KX901190	KX901244	Shen et al. 2019
*C.glaucus*	Viacheslav Spirin 5317	Russia	MG137078							Miettinen et al. 2018
*C.glaucus*	Viacheslav Spirin 6580 (holotype)	Russia	MG137081						MG137145	Miettinen et al. 2018
*C.gossypinus*	Bernard Rivoire 6658	France							MG137146	Miettinen et al. 2018
*C.hirsutus*	Cui 17083 (holotype)	China	MW182179	MW182233	MW182214	MW182197	MW191554	MW191568	MW191538	Liu et al. 2021a
*C.hirsutus*	Cui 17343	China	OL423601*	OL423611*	OL437198*	OL423623*	OL444987*	OL447001*	OL444997*	Present study
*C.hirsutus*	Cui 17342	China	OL423602*	OL423612*	OL437199*	OL423624*	OL444988*	OL447002*	OL444998*	Present study
*C.livens*	Viacheslav Spirin 8728	United States	MG137090						MG137150	Miettinen et al. 2018
*C.livens*	Otto Miettinen 17177 (holotype)	United States	MG137082						MG137147	Miettinen et al. 2018
*C.luteocaesia*	Bernard Rivoire 2605	France	MG137091							Miettinen et al. 2018
*C.magnus*	Dai 21105	China	OL423603*	OL423613*	OL437200*	OL423625*	OL444989*	OL447003*	OL444999*	Present study
*C.magnus*	Cui 16983	China	MW182180	MW182234	MW182215	MW182198	MW191555	MW191569	MW191539	Liu et al. 2021a
*C.magnus*	Otto Miettinen 10634 (holotype)	China	KC595944	KC595944					MG137151	Miettinen et al. 2018
*C.mediterraneocaesius*	LY BR 4274	France	KX900886		KX901024	KX901099				Shen et al. 2019
*C.microporus*	Cui 11014 (holotype)	China	KX900878	KX900948	KX901016	KX901091		KX901201		Shen et al. 2019
*C.microporus*	Dai 11717	China	KX900877	KX900947	KX901015	KX901090		KX901200		Shen et al. 2019
*C.nothofagicola*	Cui 16697 (holotype)	Australia	MW182181	MW182235	MW182216	MW182199	MW191556	MW191570	MW191540	Liu et al. 2021a
*C.nothofagicola*	Dai 18765	Australia	MW182182	MW182236	MW182217	MW182200	MW191557		MW191541	Liu et al. 2021a
*C.piceicola*	Cui 10626 (holotype)	China	KX900862	KX900932	KX901001	KX901075		KX901185		Shen et al. 2019
*C.piceicola*	Cui 12158	China	KX900866	KX900936	KX901004	KX901079	KX901153	KX901189	KX901243	Shen et al. 2019
*C.populi*	Otto Miettinen 17043 (holotype)	United States	MG137092						MG137153	Miettinen et al. 2018
*C.populi*	Cui 17087a	China	MW182183	MW182237	MW182218	MW182201	MW191558	MW191571	MW191542	Liu et al. 2021a
*C.populi*	Dai 18934	China	OL423604*	OL423614*	OL437201*	OL423626*	OL444990*	OL447004*	OL445000*	Present study
*C.populi*	Cui 17557	China	OL423605*	OL423615*	OL437202*	OL423627*	OL444991*	OL447005*	OL445001*	Present study
*C.rigidus*	Cui 17032 (holotype)	China	OL423606*	OL423617*	OL437204*	OL423629*	OL444993*		OL445003*	**Present study**
*C.simulans*	Otto Miettinen 20422	Finland	MG137110						MG137160	Miettinen et al. 2018
*C.simulans*	Tuomo Niemelä 8846 (holotype)	Finland	MG137103							Miettinen et al. 2018
*C.subcaesius*	Josef Vlasák 0110/24	Czech Republic	MG137117						MG137164	Miettinen et al. 2018
*C.subcaesius*	Alix David 652 (isotype)	France	MG137116							Miettinen et al. 2018
*C.subhirsutus*	Cui 11330	China	KX900873	KX900943	KX901011	KX901086		KX901196	KX901250	Shen et al. 2019
*C.subhirsutus*	Dai 14892 (holotype)	China	KX900871	KX900941	KX901009	KX901084		KX901194	KX901248	Shen et al. 2019
*C.submicroporus*	Cui 16306	China	MW182184	MW182239	MW182220	MW182203	MW191560	MW191573	MW191544	Liu et al. 2021a
*C.submicroporus*	Cui 18156 (holotype)	China	MW182186	MW182241	MW182222	MW182205		MW191574		Liu et al. 2021a
*C.subungulatus*	Cui 18046 (holotype)	China	MW448566*	MW448563*	MW448560*	MW448559*	MW452598*		**MW452603**	**Present study**
*C.subungulatus*	Zhao 10833	China	MW742586*	OL423616*	OL437203*	OL423628*	OL444992*		OL445002*	**Present study**
*C.subviridis*	Viacheslav Spirin 8774a	United States	MG137120						MG137166	Miettinen et al. 2018
*C.subviridis*	Reijo Penttilä 14376	Finland							MG137165	Miettinen et al. 2018
*C.tenuicontextus*	Cui 16280 (holotype)	China	OL423607*	OL423618*	OL437205*	OL423630*			OL445004*	**Present study**
*C.tenuicontextus*	Zhao 813	China	MG231802*	OL423619*	OL437206*	OL423631*			OL445005*	**Present study**
*C.tenuis*	Cui 10788 (holotype)	China	KX900885	KX900955	KX901023	KX901098	KX901161	KX901208		Shen et al. 2019
*C.tenuis*	Dai 12974	China	KX900884	KX900954	KX901022	KX901097	KX901160	KX901207	KX901258	Shen et al. 2019
*C.tricolor*	Cui 12233 (holotype)	China	KX900876	KX900946	KX901014	KX901089		KX901199	KX901253	Shen et al. 2019
*C.tricolor*	Cui 10790	China	KX900875	KX900945	KX901013	KX901088		KX901198	KX901252	Shen et al. 2019
*C.ungulatus*	Cui 10778	China	KX900870	KX900940	KX901008	KX901083		KX901193	KX901247	Shen et al. 2019
*C.ungulatus*	Dai 12897 (holotype)	China	KX900869	KX900939	KX901007	KX901082	KX901154	KX901192	KX901246	Shen et al. 2019
*C.yanae*	Heikki Kotiranta 27606	Russia	MG137122						MG137168	Miettinen et al. 2018
*C.yanae*	Heikki Kotiranta 27454 (holotype)	Russia	MG137121						MG137167	Miettinen et al. 2018
*Cystidiopostiahibernica*	Cui 2658	China	KX900905	KX900975	KX901045	KX901118		KX901218		Shen et al. 2019
*C.inocybe*	LY BR 3703	France	KX900903	KX900973	KX901044	KX901116			KX901267	Shen et al. 2019
*C.pileata*	Cui 10034	China	KX900908	KX900956	KX901050	KX901122	KX901170	KX901222	KX901269	Shen et al. 2019
*Fuscopostiaduplicate*	Dai 13411 (holotype)	China	KF699125	KJ684976	KR606027	KR605928	KX901174	KR610845	KR610756	Han et al. 2016
*F.fragilis*	JV 0610-8	Czech	JF950573							Vampola et al. 2014
*F.lateritia*	Dai 2652	China	KX900913	KX900983						Shen et al. 2019
*F.leucomallella*	Cui 9599	China	KF699123	KJ684983	KX901056	KX901129	KX901176	KX901228	KX901272	Shen et al. 2019
*Jahnoporusbrachiatus*	X 3232	Russia	KU165781							Spirin et al. 2015
*J.hirtus*	Spinosa 10 X 2014	United States	KU165784				KY949044			Spirin et al. 2015
*J.oreinus*	X 3241	Russia	KU165785							Spirin et al. 2015
*Oligoporusrennyi*	TN-6645	Finland	KC595929	KC595929						Ortiz-Santana et al. 2013
*O.sericeomollis*	Cui 9870	China	KX900920	KX900990	KX901068	KX901141	KX901184			Shen et al. 2019
*Osteinaobducta*	Cui 10074	China	KX900924	KX900994	KX901071	KX901144		KX901240		Shen et al. 2019
*O.undosa*	Dai 7105	China	KX900921	KX900991	KX901069	KX901142		KX901238		Shen et al. 2019
*Postiaamurensis*	Dai 903 (holotype)	China	KX900901	KX900971	KX901042					Shen et al. 2019
*P.hirsuta*	Cui 11237 (holotype)	China	KJ684970	KJ684984	KX901038	KX901113			KX901266	Shen et al. 2019
*P.lactea*	Cui 12141	China	KX900892	KX900962	KX901029	KX901104	KX901163	KX901211	KX901260	Shen et al. 2019
*P.lowei*	Cui 9585	China	KX900898	KX900968	KX901035	KX901110				Shen et al. 2019
*P.ochraceoalba*	Cui 10802 (holotype)	China	KM107903	KM107908	KX901041	KX901115		KX901216		Shen et al. 2015
*P.sublowei*	Cui 9597 (holotype)	China	KX900900	KX900970	KX901037	KX901112			KX901265	Shen et al. 2019
*P.tephroleuca*	Dai 12610	Finland	KX900897	KX900967	KX901034	KX901109	KX901166	KX901214	KX901263	Shen et al. 2019
*Spongiousgloeoporus*	Cui 10401	China	KX900915	KX900985	KX901060	KX901133		KX901232		
*S.floriformis*	Cui 10292	China	KM107899	KM107904	KX901058	KX901131	KX901178	KX901230	KX901274	
*S.floriformis*	Dai 13887	China	KX900914	KX900984	KX901057	KX901130	KX901177	KX901229	KX901273	

*Newly generated sequences for this study. New species are shown in bold.

In the phylogenetic trees, *Cyanosporusflavus* grouped together with *C.fusiformis*, *C.subungulatus* and *C.ungulatus* (Figs 2, 3). *Cyanosporusfusiformis* differs from *C.flavus* by having white to cream pileal surface when fresh, clay-buff pore surface when dry and larger pores (4–5 per mm) and by growing on angiosperm woods (Shen et al. 2019); *C.subungulatus* differs from *C.flavus* in its glabrous pileal surface, cream to pinkish buff pore surface when dry and wider basidiospores (4.5–5.2 × 1.1–1.4 μm); *C.ungulatus* differs from *C.flavus* by having ungulate basidiomata, sulcate pileal surface with olivaceous buff, pinkish buff, cream to ash-grey and white zones when fresh (Shen et al. 2019). *Cyanosporushirsutus* and *C.subhirsutus* have pileate basidiomata with hirsute, blue tint to the pileal surface and slightly thick-walled basidiospores like *C.flavus*, but *C.hirsutus* differs by having wider basidiospores (4–4.7 × 1.2–1.5 μm; Liu et al. 2021a), while *C.subhirsutus* has larger pores (2–3 per mm; Shen et al. 2019). Besides, *C.hirsutus* and *C.subhirsutus* are distant from *C.flavus* in the phylogenetic analyses (Figs 2, 3). *Cyanosporussubungulatus* and *C.ungulatus* share similar pores and basidiospores; however, *C.ungulatus* differs by having ungulate basidiomata, glabrous and sulcate pileal surface, narrower context hyphae and tramal hyphae (Shen et al. 2019).

Phylogenetically, *Cyanosporusrigidus* form a separate lineage different from other species in the genus. Morphologically, *C.submicroporus* share similar pores and basidiospores with *C.rigidus*, but *C.submicroporus* differs by having cream to pinkish buff pileal surface and white to smoke grey pore surface when fresh, buff to buff-yellow pileal surface and buff to olivaceous buff pore surface when dry. *Cyanosporusauricomus* and *C.luteocaesius* resemble *C.rigidus* in morphology by producing yellow-colored basidiomata, but *C.auricomus* differs from *C.rigidus* by having a hirsute pileal surface and larger basidiospores (4.4–5.6 × 1.5–1.8 μm; Miettinen et al. 2018); *C.luteocaesius* differs from *C.rigidus* by having larger pores (3–5 per mm) and basidiospores (4.3–6.1 × 1.5–1.9 μm; Miettinen et al. 2018).

Phylogenetically, *C.tenuicontextus* is closely related to *C.caesiosimulans*, *C.cyanescens*, *C.populi*, *C.subviridis* and *C.yanae* (Figs 2, 3). Morphologically, they share similar pores; but *C.caesiosimulans* differs by having larger basidiospores (4.2–5.5 × 1.1–1.4 μm), and a wide distribution area (Europe and North America; Miettinen et al. 2018); *C.cyanescens* differs in having light bluish-greyish tint in older and dry specimens and larger basidiospores (4.7–6.1 × 1.1–1.6 μm; Miettinen et al. 2018); *C.populi* differs in its larger basidiospores (4.2–5.6 × 1–1.3 μm), and a wide distribution area (East Asia, Europe and North America; Miettinen et al. 2018; Liu et al. 2021a); *C.subviridis* differs in its conchate basidiomata, distributed in Europe and North America and grows only on gymnosperms (*Abies* sp., *Picea* sp. and *Pinus* sp.; Miettinen et al. 2018); *C.yanae* differs by having narrower generative hyphae (3–4 μm in context, 2.2–2.9 μm in tubes), larger basidiospores (4.3–5.8 × 1.2–1.6 μm), distributed in Europe and grows only on gymnosperm (*Larix* sp., *Pinus* sp.; Miettinen et al. 2018). *Cyanosporusbifarius* is also distributed in Lanping County, Yunnan Province of China, they share similar pores and basidiospores, but *C.bifarius* grows only on gymnosperm trees (*Picea* sp., *Pinus* sp., *Larix* sp.; Miettinen et al. 2018), and *C.bifarius* is distant from *C.tenuicontextus* in the phylogenetic analyses (Figs 2, 3).

The natural distribution of plant-associated fungi across broad geographic ranges is determined by a combination of the distributions of suitable hosts and environmental conditions (Lodge 1997; Brandle and Brandl 2006; Gilbert et al. 2007, 2008). Species in *Cyanosporus* have a wide distribution range (Asia, Europe, North America, South America and Oceania; Table 2) and variable host type (angiosperms and gymnosperms). As for distribution ranges, 23 species of *Cyanosporus* are distributed in Asia, 16 species in Europe, seven species in North America, one species in South America and one species in Oceania. As for host trees, nine species of *Cyanosporus* grow only on angiosperm trees, 15 species only on gymnosperm trees, and eleven species both on angiosperm and gymnosperm trees (Table 1). In some cases, some *Cyanosporus* species have host specificity, at least regionally, such as in Europe, *C.auricomus* only growth on *Pinussylvestris*, *C.cyanescens* only growth on *Piceaabies*, *C.populi* prefers *Populustremula*, and *C.luteocaesia* have been recorded only from *Pinus* sp. (Miettinen et al. 2018).

In the current study, 77 samples of *Cyanosporus* throughout China and 11 samples outside of China have been morphologically examined in detail. The specimens collected from China representing 21 species were sequenced here and referred to in our phylogeny, viz., *C.alni*, *C.auricomus*, *C.bifarius*, *C.bubalinus*, *C.coeruleivirens*, *C.comatus*, *C.flavus*, *C.fusiformis*, *C.hirsutus*, *C.magnus*, *C.microporus*, *C.piceicola*, *C.populi*, *C.rigidus*, *C.subhirsutus*, *C.submicroporus*, *C.subungulatus*, *C.tenuicontextus*, *C.tenuis*, *C.tricolor* and *C.ungulatus.* Another two species reported in a previous study, viz., *C.glauca* (=*Postiaglauca* Spirin & Miettinen) and *C.simulans* (=*Postiasimulans* (P. Karst.) Spirin & Rivoire; Miettinen et al. 2018) were also found from China. Among these *Cyanosporus* species, 15 are endemic to China so far, viz., *C.bubalinus*, *C.flavus*, *C.fusiformis*, *C.hirsutus*, *C.microporus*, *C.piceicola*, *C.rigidus*, *C.subhirsutus*, *C.submicroporus*, *C.subungulatus*, *C.tenuicontextus*, *C.tenuis*, *C.tricolor* and *C.ungulatus*. The *Cyanosporus* species formed a distribution center in Southwest China. This may be due to the complex and diverse ecological environment and diverse host trees in this region, which provide a rich substrate for the growth of *Cyanosporus* species. The geographical locations of the *Cyanosporus* species distributed in China are indicated on the map (Fig. 1).

In summary, we performed a comprehensive study on the species diversity and phylogeny of *Cyanosporus* with an emphasis on Chinese collections. So far, 35 species are accepted in the *Cyanosporus* around the world, including 23 species from China. Currently, *Cyanosporus* is characterized by an annual growth habit, resupinate to effused-reflexed or pileate, soft corky, corky, fragile to hard corky basidiomata, velutinate to hirsute or glabrous pileal surface with blue-tinted, white to cream or yellow-colored, white to cream pore surface with round to angular pores, a monomitic hyphal system with clamped generative hyphae, and hyaline, thin- to slightly thick-walled, smooth, narrow, allantoid to cylindrical basidiospores that are usually weakly cyanophilous; it grows on different angiosperm and gymnosperm trees, causes a brown rot of wood and has a distribution in Asia, Europe, North America, Argentina in South America and Australia in Oceania (McGinty 1909; Shen et al. 2019; Liu et al. 2021a).

## Supplementary Material

XML Treatment for Cyanosporus flavus

XML Treatment for Cyanosporus rigidus

XML Treatment for Cyanosporus subungulatus

XML Treatment for Cyanosporus tenuicontextus
